# Ruxolitinib for pediatric acute and chronic graft-versus-host disease: a single-center retrospective study of efficacy and safety

**DOI:** 10.1007/s00277-025-06225-0

**Published:** 2025-02-04

**Authors:** Chiao-Yu Cheng, Meng-Yao Lu, Hsiu-Hao Chang, Yung-Li Yang, Chang-Hsueh Wu, Dong-Tsamn Lin, Kai-Hsin Lin, Shu-Wei Chou, Shiann-Tarng Jou

**Affiliations:** 1 Department of Pediatrics, National Taiwan University Hospital, National Taiwan University College of Medicine, No. 8 Chung-Shan South Road, Taipei, 10041 Taiwan; 2 Graduate Institute of Clinical Medicine, National Taiwan University College of Medicine, Taipei, Taiwan; 3 Department of Laboratory Medicine, National Taiwan University Hospital, National Taiwan University College of Medicine, Taipei, Taiwan; 4 Department of Pharmacology, National Taiwan University Hospital, National Taiwan University College of Medicine, Taipei, Taiwan

**Keywords:** Ruxolitinib, Acute graft-versus-host disease, Chronic graft-versus-host disease, Pediatrics, Allogeneic hematopoietic stem cell transplantation

## Abstract

Graft-versus-host disease (GVHD) is a major concern for patients undergoing allogeneic hematopoietic stem cell transplantation (HSCT). Ruxolitinib has been proven effective in treating adult steroid-refractory GVHD; however, studies on pediatric patients are relatively scarce. Thus, this single-center study evaluated the efficacy and safety of ruxolitinib in pediatric patients with steroid-refractory GVHD. We retrospectively reviewed the data of patients aged < 18 years who underwent allogeneic HSCT and received ruxolitinib treatment for either acute GVHD (aGVHD) or chronic GVHD (cGVHD) between 2018 and 2023. Data on the clinical response, concomitant and subsequent medications, adverse events, and outcomes were obtained through medical chart review. Sixteen patients were analyzed in this study: seven with aGVHD and nine with cGVHD. The overall response rate for the 16 patients was 81% (aGVHD, 86%; cGVHD, 77%). The overall survival rate was 56%: (aGVHD, 57%; cGVHD, 55%). For 11 patients with at least stable disease, steroid dosage could be reduced by at least 75%; however, corticosteroids were successfully tapered off in only six patients at the last follow-up. Among four patients with documented lung cGVHD, none experienced lung cGVHD progression at 1-year follow-up. Further, 50% of the patients experienced grade 3 or 4 neutropenia and/or thrombocytopenia, and 56% had viral reactivation. Two patients discontinued ruxolitinib owing to adverse events. Ruxolitinib treatment for pediatric patients with aGVHD and cGVHD is associated with a high overall response rate, significant steroid-sparing effect, acceptable toxicity, and manageable adverse events. However, blood count and viral reactivation should be closely monitored during ruxolitinib use.

## Introduction

Hematopoietic stem cell transplantation (HSCT) has been used to manage various malignant and non-malignant diseases in pediatric patients. Despite gaining experience in HSCT, graft-versus-host disease (GVHD), including acute GVHD (aGVHD) and chronic GVHD (cGVHD), remains a challenge after HSCT.

The pathophysiology of aGVHD can be divided into three phases [1]. First, host tissues are damaged by underlying diseases, treatments, infections, and the conditioning regimen. Second, cytokines released by damaged tissues activate host antigen-presenting cells, which further activate mature donor T cells. Finally, effector T cells and inflammatory cytokines attack the epithelial cells of the skin, liver, and gastrointestinal tract, causing aGVHD. Similarly, the pathophysiology of cGVHD can be divided into three phases [1]. First tissue damage occurs, which is caused by the conditioning regimen, preceding aGVHD, and infections. Second, the tissue damage leads to the release of inflammatory mediators and activation of immune effector cells. Finally, fibrogenic peptides initiate fibroblast activation and the production of extracellular matrix collagen, causing sclerotic phenotype. Various cytokines are involved in the pathophysiology of aGVHD and cGVHD.

The first-line systemic therapy for aGVHD and cGVHD is glucocorticoids [2]. However, some patients remain resistant or refractory to glucocorticoids. Furthermore, prolonged glucocorticoid use results in various well-known adverse effects in children, including infection due to pronounced immunosuppression, growth suppression, and osteoporosis [3]. Therefore, other potential treatments for steroid-refractory GVHD are required, particularly for the pediatric population.

Janus kinase (JAK) is part of the receptor complex of various cytokines, such as interleukin (IL)-2, IL-4, IL-7, IL-9, IL-15, and IL-21 [4]. These cytokines activate the JAK molecule and induce the binding of the signal transducers of activated transcription (STAT) protein, which is activated through auto-phosphorylation [1, 4]. The activation of the JAK–STAT pathway is crucial to the activation, survival, and lineage commitment of T cells; the activation of neutrophils; and the differentiation and maturation of dendritic cells. Ruxolitinib, a selective inhibitor of JAK proteins—particularly JAK1 and JAK2—ameliorates the inflammatory state of different immune-mediated diseases and has been used as an anticancer therapy. Through JAK1/JAK2 inhibition, ruxolitinib plays the role of controlling aGVHD and cGVHD through decreased neutrophil migration, cytokine signaling blockade, and inhibition of cytokine release and T-cell expansion [5].

Ruxolitinib has been proven efficacious in controlling aGVHD and cGVHD in REACH2 and REACH3 trials, respectively [6, 7]. However, these trials included only patients aged > 12 years. Studies on the efficacy of ruxolitinib and its associated adverse events in the pediatric population are relatively scarce.

Therefore, in this study, we aimed to evaluate the efficacy of ruxolitinib and its associated adverse events in pediatric patients with aGVHD or cGVHD.

## Materials and methods

### Patients

Patients aged < 18 years who were receiving ruxolitinib for aGVHD or cGVHD after allogeneic HSCT were included in this single-center study. The diagnosis and grading of aGVHD and cGVHD were based on previous reports and criteria published by the National Institute of Health [8, 9]. The dose of ruxolitinib is given according to REACH4 and REACH5 study designs [10, 11]. Patients receiving ruxolitinib for indications other than GVHD, such as hemophagocytic lymphohistiocytosis or Philadelphia-like acute lymphoblastic leukemia, were excluded from the analysis. Clinical data, including baseline demographics, severity and extent of GVHD, medications before or after ruxolitinib use, and clinical responses, were collected retrospectively, with the approval of the Institutional Review Board.

### Response assessment

For patients with aGVHD, ruxolitinib use duration, response on day 28, best overall response, corticosteroid dose reduction on day 56, and grade ≥ 3 adverse effects and infections that occurred during ruxolitinib use were recorded to evaluate treatment response. For patients with cGVHD, ruxolitinib use duration, corticosteroid dose reduction up to week 24, best overall response, organ response, grade ≥ 3 adverse effects, and infections that occurred during ruxolitinib use were recorded to assess treatment response.

Clinical response was defined based on previous articles and criteria published by the National Institute of Health [9, 12]. In cases of aGVHD, complete response (CR) was defined as complete resolution of aGVHD symptoms in all organs, whereas partial response (PR) was defined as improvement in stage in all initially involved organs without complete resolution and worsening in any other target organs. In cases of cGVHD, CR was defined as the absence of cGVHD manifestations, whereas PR was defined as clinical improvement in one or more organs without progression in any other target organs. The Common Terminology Criteria for Adverse Events was used to evaluate adverse events.

### Statistical analysis

Continuous variables are presented as medians and ranges, whereas categorical variables are presented as percentages. Statistical analysis was performed using SPSS version 22.0 (Chicago, IL, USA).

## Results

### Patients

In total, 16 patients received ruxolitinib for aGVHD (*n* = 7) or cGVHD (*n* = 9) after allogeneic HSCT.

Of the seven patients who received ruxolitinib for aGVHD, four were males, and three were females. Their median age was 6.3 years (range: 0.7–16.5 years). The indication of allogenic HSCT, donor type, conditioning regimen and severity of aGVHD are presented in Table 1 while the use of GVHD medication is shown in Table 2. All patients received cyclosporine and methotrexate as GVHD prophylaxis regimen. Grades 2, 3, and 4 aGVHD were diagnosed in four, one, and two patients, respectively. Both patients with grade 4 aGVHD had stage 4 lower gastrointestinal disease. Two patients developed aGVHD after peripheral blood stem cell boost. Six patients were receiving corticosteroid treatment at the time of ruxolitinib initiation, whereas one had discontinued corticosteroid treatment before ruxolitinib use owing to post-transplant lymphoproliferative disorder (PTLD). Two patients received tacrolimus and sirolimus before ruxolitinib, and two received budesonide. The two patients who received budesonide before ruxolitinib received etanercept after ruxolitinib due to GVHD progression.

**Table 1:** Demographics of patients with acute GVHD

No.	Age (years)	Indication	Donor type	Conditioning	aGVHD before Ruxolitinib			
					Skin	Liver	GI	Overall
1	2.9	Atypical SCID	MMUD	BuFlu	3	2	4	4
2	16.2	ALD	MMUD	BuCy	3	0	1	2
3	4.9	JMML	MMUD	FluCyEto	0	2	1	3
4	5.1	ALD	Haplo	BuCy	3	0	1	2
5	6.6	SAA	MMUD	FluCyATG	3	0	1	2
6	0.6	CGD	MMUD	BuFluCy	3	1	1	2
7	11.1	ALL	Haplo	TBICy	1	1	4	4

ALD: Adrenoleukodystrophy; ALL: Acute lymphoblastic leukemia; ATG: Anti-thymocyte globulin; Bu: Busulfan; CGD: Chronic granulomatous disease; CsA: Cyclosporine; Cy: Cyclophosphamide; Eto: Etoposide; Flu: Fludarabine; GI: gastrointestinal; GVHD: Graft-versus-host disease; Haplo: Haploidentical; JMML: Juvenile myelomonocytic leukemia; MMUD: Mismatched unrelated donor (human leukocyte antigen ≤ 9/10 matched); SAA: Severe aplastic anemia; SCID: Severe combined immunodeficiency; TBI: Total body irradiation

**Table 2:** Treatment and outcomes of patients with acute GVHD

No.	Other GVHD medication	Ruxolitinib dose	Duration of treatment (days)	Best OR	Adverse effects / Infection	Survival
1	Steroid, CsA, Budesonide, Etanercept^a^	2.5 mg BID	646	CR	Gr.3 Neutropenia CMV, UTI	Alive
2	Steroid, CsA	5 mg BID	149	CR	Gr. 4 Neutropenia Gr. 3 Thrombocytopenia CMV, EBV	Dead
3	Steroid, CsA, Tacrolimus, Sirolimus	5 mg BID	1372	CR	Gr. 3 Neutropenia Gr. 4 Thrombocytopenia	Alive
4	Steroid, CsA	10 mg BID	159	CR	Nil	Dead
5	Steroid, CsA	5 mg QD	57	CR	CMV	Alive
6	Steroid, CsA	1.25 mg QD	93	CR	Gr. 3 Neutropenia Gr. 4 Thrombocytopenia	Dead
7	Steroid, CsA, Tacrolimus, Sirolimus, Budesonide, Etanercept^a^	5 mg BID	56	PD	Gr. 4 Neutropenia Gr. 3 Thrombocytopenia BK virus	Dead

^a^ Indicates medication added after ruxolitinib

CMV: Cytomegalovirus; CR: Complete response; CsA: Cyclosporine; EBV: Epstein-Barr virus; OR: Overall response; PD: Progressive disease; UTI: Urinary tract infection

Among the nine patients who received ruxolitinib for cGVHD, five were males, and four were females. Their median age was 9.0 years (range: 2.7–17.9 years). The indication of allogenic HSCT, donor type, conditioning regimen and severity of cGVHD are presented in Table 2. Regarding the severity of cGVHD, three of the nine patients had moderate cGVHD, whereas six had severe cGVHD. Four patients experienced lung cGVHD, with one having a score of 1 and three having a score of 3. All patients received cyclosporine and methotrexate as GVHD prophylaxis regimen. Before initiation of ruxolitinib, all nine patients received corticosteroid, eight received cyclosporine, five received tacrolimus, four received sirolimus, one received mycophenolate mofetil, and one received etanercept. Six of the nine patients were still receiving corticosteroids at the time of ruxolitinib initiation. Under use of ruxolitinib, one patient added sirolimus and one received basiliximab for further control of chronic GVHD.

**Table 3:** Demographics of patients with chronic GVHD

No.	Age (year)	Underlying disease	Donor Type	Conditioning	cGVHD before Ruxolitinib	
					Organ & Score	Overall
1	1.5	MPS	CB	BuCy	Skin 2, GI 1	Moderate
2	1.7	JMML	CB	BuCyMel	Skin 2, GI 1	Moderate
3	13.7	AML	MMUD	BuCy	Skin 2, Liver 3, Eye 2, Mouth 1	Severe
4	7.5	SAA	Sibling	CyATG	Skin 2, Lung 3, Eye 1, Mouth 1	Severe
5	17.4	ALL	MMUD	TBICy	Skin 1, Lung 1, GI 1	Moderate
6	9.7	AML	Haplo	FluBuCy	Skin 3, Liver 1, GI 2, Mouth 3	Severe
7	10.4	AML	Haplo	BuCy	Lung 3	Severe
8	1.5	AML	Haplo	BuCy	Skin 2, Liver 2, GI 3	Severe
9	4.6	LAD	Haplo	FluMel	Skin 2, Lung 3	Severe

ALL: Acute lymphoblastic leukemia; AML: Acute myeloid leukemia; ATG: Anti-thymocyte globulin; Bu: Busulfan; CB: Cord blood; CsA: Cyclosporine; Cy: Cyclophosphamide; Flu: Fludarabine; GI: gastrointestinal; GVHD: Graft-versus-host disease; Haplo: Haploidentical; JMML: Juvenile myelomonocytic leukemia; LAD: Leukocyte adhesion deficiency; Mel: Mephalan; MMUD: Mismatched unrelated donor(human leukocyte antigen ≤ 9/10 matched); MPS: Mucopolysaccharidoses; SAA: Severe aplastic anemia; TBI: Total body irradiation

**Table 4:** Treatment and outcome of patients with chronic GVHD

No.	Other GVHD medication	Ruxolitinib dose	Treatment duration (day)	Best OR	Organ response	Adverse effect / Infection	Survival
1	Steroid, CsA, Tacrolimus, Sirolimus	5 mg QD	1296	CR	Skin / GI: CR	Nil	Alive
2	Steroid, CsA, Tacrolimus, Sirolimus	5 mg BID	661	CR	Skin / GI: CR	Gr. 3 Neutropenia	Alive
3	Steroid, CsA Sirolimus^a^	5 mg BID	41	PR	skin / eye: PR liver/mouth: CR	Gr. 4 Triglyceridemia Gr. 4 Pancreatitis HSV, Fungal infection	Dead
4	Steroid, CsA Tacrolimus	5 mg BID	790	PR	Lung: SD Skin: PR eye/mouth: CR	Nil	Alive
5	Steroid, CsA, Tacrolimus	10 mg BID	113	CR	Skin / GI: CR Lung: CR	Gr. 4 Neutropenia Gr. 4 Thrombocytopenia EBV(CSF)	Alive
6	Steroid, CsA, Tacrolimus, Sirolimus	5 mg BID	51	PR	skin: PR mouth/GI: PR Liver: CR	CMV	Dead
7	Steroid, CsA, MMF	10 mg BID	1458	Unchanged	Unchanged	Nil	Dead
8	Steroid, CsA, Sirolimus, Etanercept Basiliximab^a^	5 mg QD	21	Unchanged	Unchanged	Gr. 5 Sepsis EBV	Dead
9	Steroid, CsA	5 mg QD	323	PR	Lung: SD Skin: PR	Nil	Alive

^a^ Indicates medication added after ruxolitinib

CMV: Cytomegalovirus; CR: Complete response; CsA: Cyclosporine; CSF: Cerebrospinal fluid; EBV: Epstein-Barr virus; GI: gastrointestinal; HSV: Herpes simplex virus; MMF: Mycophenolate mofetil; OR: Overall response; PR: Partial response; SD: Stable disease

### Clinical response

The treatment details and outcome of patients are concluded in Tables 3 and 4. In the aGVHD group, five patients achieved CR, one achieved PR, and one had progressive disease (PD) on day 28. One of the patients who achieved CR was not receiving corticosteroid treatment at the time of ruxolitinib initiation. In three of the other four patients who achieved CR, corticosteroids were successfully discontinued before day 56. For the only patient who was still receiving corticosteroids after starting ruxolitinib, a 90% reduction in corticosteroid dosage was achieved on day 56. Five of the seven patients who received ruxolitinib for aGVHD kept receiving ruxolitinib as the final treatment for aGVHD, whereas one who achieved PR and one with PD received etanercept for better control of aGVHD after ruxolitinib. On day 56, six patients achieved CR, and the patient with PD on day 28 still had PD. Six of the seven patients achieved CR as their best overall response. Three patients were alive at the latest follow-up, whereas two died from infection, one from thrombotic microangiopathy, and one from idiopathic pneumonia syndrome.

In the cGVHD group, three patients achieved CR, four achieved PR, and the cGVHD status of two patients remained unchanged as the best overall response. At week 24, two patients achieved CR, two achieved PR, and one had stable disease. Ruxolitinib use was discontinued in four patients before week 24. The 1-year treatment response was the same as that at week 24. No patient experienced PD after ruxolitinib initiation. All three patients with moderate cGVHD achieved CR. For specific organ response, among the seven patients with skin involvement, three achieved CR, whereas four achieved PR. Among the four patients with gastrointestinal involvement, three achieved CR, whereas one achieved PR. Notably, among the four patients with lung involvement, only one achieved CR, whereas the lung condition remained unchanged in the remaining three patients at 1 year. Of the six patients still receiving corticosteroids at the time of ruxolitinib initiation, corticosteroids were discontinued for three within 6 months. For the remaining three, corticosteroids were reduced by 75%, 87.5%, and 90% relative to their original corticosteroid dosage. To alleviate cGVHD, one patient who achieved PR received sirolimus after ruxolitinib, and one with SD received basiliximab after ruxolitinib. Five of the nine patients with cGVHD were alive at the latest follow-up, whereas one died from lung cGVHD, two from infection, and one from underlying disease progression.

### Adverse events

Cytopenia and viral infection were the two most common adverse events in our cohort. High-grade (grade 3/4) cytopenia occurred in eight patients (50%) in our cohort, with five experiencing both grade 3/4 neutropenia and thrombocytopenia. Two patients had grade 3 neutropenia, and one had grade 4 thrombocytopenia. Patients with high-grade cytopenia continued treatment with dose adjustment, whereas only one patient discontinued treatment owing to grade 4 thrombocytopenia.

Viral infection occurred in nine patients (56%): five were infected with cytomegalovirus (CMV), three with Epstein–Barr virus (EBV), and one with BK virus. Notably, most of the viral infections that occurred in our patients were manageable, except for one patient with EBV reactivation, who was later diagnosed with central nervous system PTLD and discontinued ruxolitinib. In addition to cytopenia and viral infection, grade 4 hypertriglyceridemia and pancreatitis were also reasons for which ruxolitinib was discontinued for one patient.

## Discussion

In the present study, we demonstrated a high overall response rate (ORR) and CR rate for pediatric patients with GVHD treated with ruxolitinib. This is consistent with the results of previous studies on the subject worldwide (Table 3). According to the literature, the ORR for patients with aGVHD receiving ruxolitinib ranged between 45% and 87%, while an ORR of 86% was observed in the present study. Furthermore, the ORR for patients with cGVHD receiving ruxolitinib ranged between 70% and 91% in previous studies, while an ORR of 77% was observed in the present study. The steroid-sparing effect in the present study was also promising, with median steroid dose reduction being 100% in the aGVHD group on day 56 and 95% in the cGVHD group at 6 months. Among 11 patients with SD, corticosteroids were successfully tapered off for six at the last follow-up.

**Table 5:** Pediatric experience of ruxolitinib use in this study and similar recent literature

	SR-aGVHD			SR-cGVHD (including overlap syndrome)		
	Patients	ORR	CR	Patients	ORR	CR
Khandelwal et al., 2017	11	45%	9%	-	-	-
González Vicent et al., 2019	13	77%	31%	9	89%	22%
Uygun et al., 2020	13	77%	69%	16	81%	6%
Laisne et al., 2020	29	72%	66%	-	-	-
Yang et al., 2021	17	65%	30%	36	81%	28%
Mozo et al., 2021	8	87%	38%	12	91%	8%
Wang et al., 2022	-	-	-	20	70%	10%
NTUCH cohort	7	86%	86%	9	77%	33%

aGVHD: Acute graft-versus-host disease; cGVHD: Chronic graft-versus-host disease; SR: Steroid-refractory; ORR: overall response rate; CR: complete response

Ruxolitinib has been proven efficacious in controlling glucocorticoid-refractory aGVHD in REACH2 trial. In the REACH2 trial, 309 patients were enrolled, with 154 randomly assigned to the ruxolitinib group and 155 to the control group [6, 13]. The cumulative incidence of loss of response at 6 months was 10% and 39% in the ruxolitinib and control groups, respectively. The median failure-free survival was longer with ruxolitinib than with the control therapy (5.0 months vs. 1.0 month).

In the present study, the high ORR in the aGVHD group might be explained by the disease status of patients being relatively non-severe, with only one patient having grade 4 aGVHD. However, despite stage 4 gastrointestinal involvement, the patient was responsive to ruxolitinib and achieved CR as the best overall response. According to previous studies, GVHD with gastrointestinal involvement was not only associated with poor response rate to traditional aGVHD treatment and high non-relapse mortality rate after allogenic HSCT but was also indicated as one of the major risk factors for corticosteroid resistance [14, 15]. In the REACH2 trial, most enrolled patients had lower gastrointestinal aGVHD (68.3%), and the overall response in the trial was also confirmative [6]. Furthermore, according to a recent systematic review, in a cohort of patients aged < 12 years, ORRs of 72% and 69% were observed among patients with grade 3/4 and gastrointestinal aGVHD, respectively, with no significant difference in response according to grading and gastrointestinal involvement [15]. Therefore, ruxolitinib may also be effective in treating grade 4 gastrointestinal aGVHD. Notably, according to a previous report, the time to best overall response may be longer than 28 days [4]. In the present study, five of seven patients with gastrointestinal aGVHD achieved CR within 28 days and one patient had PR on day 28 only but achieved CR afterward.

In the REACH3 trial, 329 patients were enrolled, with 165 randomly assigned to the ruxolitinib group and 164 to the control group [7, 13]. The ORR at the primary endpoint was significantly higher with ruxolitinib than with the control therapy (49.7% vs. 25.6%). The steroid dosage was reduced in both groups, with a slightly greater reduction observed in the ruxolitinib group. The probability of a maintained response at 12 months was 68.5% and 40.3% in the ruxolitinib and control groups, respectively. The median failure-free survival was longer in the ruxolitinib group than in the control group (> 18.6 months vs. 5.7 months).

In the cGVHD group in the present study, all three patients with moderate disease achieved CR, and no case of PD was observed. Among the four patients with lung cGVHD, one with a score of 1 achieved CR, the status of one with a score of 3 remained unchanged for at least 1.5 years, one with a score of 3 initially experienced a slight improvement in the forced expiratory volume over the first second, but the improvement did not fulfill PR criteria, and the status of one with a score of 3 remained unchanged for 3 years but progressed to respiratory failure afterward. Bronchiolitis obliterans syndrome is the pulmonary involvement of cGVHD and can be life-threatening. In the REACH3 trial, the response rate of patients with cGVHD with lung involvement was low, with 8.6% and 6.1% observed in the ruxolitinib and control groups, respectively [7]. However, responsiveness to ruxolitinib treatment in patients with lung cGVHD [16], particularly at an early use, has been reported in previous studies [17]. The findings in our cohort also support the role of ruxolitinib in treating lung cGVHD if administrated in the early phase. Nevertheless, further prospective studies are needed to validate this finding.

In the REACH2 and REACH3 trials, the adverse events that were more significant in the ruxolitinib group than in the control group included cytopenia, hepatotoxicity, and dyslipidemia, whereas no significant difference in CMV and EBV reactivation was observed.

The incidence of viral infection was 56% in our cohort, which was relatively higher than that in previous studies. Five patients experienced CMV reactivation. However, there was no CMV-related mortality or morbidity after appropriate treatments, including tapering down immunosuppressants or ganciclovir use. Notably, one patient developed EBV-related central nervous system PTLD, which resulted in ruxolitinib discontinuation. In recent studies, only a few cases of PLTD after ruxolitinib use have been reported [18, 19]. The causal relationship between PTLD and ruxolitinib is difficult to define owing to the use of multiple immunosuppressive agents. Ruxolitinib initiation has been reported to be a significant poor prognostic factor for the first CMV and EBV reactivation in cases of GVHD [20]. Considering the efficacy of ruxolitinib in controlling steroid-refractory GVHD, applications of novel viral prophylactic strategies (such as letermovir), and relatively rare virus-related end-organ diseases developed if viral reactivation was managed appropriately, ruxolitinib could still be used under regular viral load monitor.

Hematological adverse events were also significant, occurring in 50% of patients in our cohort. However, several possible etiologies of hematological toxicity exist, making it difficult to clarify the causality. To address the ruxolitinib-related cytopenia, ruxolitinib was continued for most patients with dose adjustment, except one patient with grade 4 thrombocytopenia.

In addition to viral infection and hematological adverse effects, grade 4 hypertriglyceridemia and pancreatitis were the reasons why ruxolitinib was discontinued for one patient. Grade 4 hypertriglyceridemia may occur due to the concomitant use of glucocorticoids (2 mg/kg/day) and voriconazole, thus inducing a drug–drug interaction. Since ruxolitinib is primarily metabolized by cytochrome P450 3A4, it is crucial to closely monitor adverse events and dose adjustment might be needed [21]. No liver toxicity was observed in the present study.

Integrating the results of recently published studies on the pediatric experience of ruxolitinib use for steroid-refractory GVHD, the ORR was generally good. A significant steroid-sparing effect was observed among responders. Generally, the adverse effects included cytopenia, viral reactivation (particularly CMV viremia), and liver toxicity. The actual frequency of adverse effects varied among different studies. The frequency of liver toxicity owing to ruxolitinib use is lower in the pediatric population than in adults [21].

Body weight and metabolism differ among children at different stages and ages; therefore, the suitable dosage for pediatric patients is under discussion. Cytochrome P450 3A4 is the primary enzyme responsible for ruxolitinib metabolism [21]. In pediatric patients, the liver is immature, and may further impact ruxolitinib metabolism. In a recent study by Cook et al., the mean ruxolitinib clearance was higher in children aged < 2 years than in those aged > 2 years, and the clearance was reduced with combined treatment with azoles and azithromycin [22]. With a higher clearance, the plasma concentration was lower. Therefore, even though the recommended pediatric dosage of ruxolitinib has been established, clinical response and adverse events should be monitored, and dose titration might be considered if suboptimal clinical response is observed, particularly in patients aged < 2 years.

Currently, the REACH4 trial is being conducted to explore the pediatric use of ruxolitinib for aGVHD [10]. The trial involves patients aged between ≥ 2 and < 18 years. The enrolled patients were distributed into three groups according to age, with different groups assigned different ruxolitinib dosages: group 1 included patients aged between ≥ 12 and < 18 years receiving 10 mg BID, group 2 included those aged between ≥ 6 and < 12 years receiving 5 mg BID, and group 3 included those aged between ≥ 2 and < 6 years receiving 4 mg/m^2^ BID. The preliminary results showed ORRs of 83.3%, 83.3%, and 86.7% on day 28 in groups 1, 2, and 3, respectively, with a general ORR of 84.4%. The durable ORR on day 56 for all patients was 66.7%, with groups 1, 2, and 3 having durable ORRs of 55.6%, 75%, and 73.3%, respectively, on day 56.

In addition to the REACH4 trial, the REACH5 trial is also underway and will be conducted to study the results of pediatric use of ruxolitinib for cGVHD. In the published interim analysis, 45 patients who underwent allogeneic HSCT and had been diagnosed with moderate-to-severe cGVHD were enrolled. The allocation of enrolled patients was similar to that in the REACH4 trial, which was according to age and ruxolitinib dosage. The interim analysis of the REACH5 trial revealed an ORR of 40%, with ORRs of 41% and 39% observed among treatment-naive and corticosteroid-refractory patients, respectively. The most common treatment-related adverse events were neutropenia and thrombocytopenia [11]. The upcoming final results of the REACH4 and REACH5 trials are highly anticipated.

Our study has some limitations. As this is a single-center study, the case number is limited. In addition, the clinical data were collected retrospectively which may lead to selection bias or information bias.

In conclusion, ruxolitinib use for GVHD after allogenic HSCT was associated with high ORR in our cohort, particularly in the aGVHD group. The dosage of corticosteroids could be reduced significantly after ruxolitinib use. Blood cell counts and CMV/EBV infection status should be closely monitored given the high rate of severe cytopenia and viral reactivation. Complete data on the pediatric use of ruxolitinib for GVHD remains lacking. Therefore, further research and discussion are required to provide a more comprehensive understanding of ruxolitinib use in pediatric patients with GVHD.

## Data Availability

No datasets were generated or analysed during the current study.
